# Case Report: Radical nephroureterectomy after toripalimab plus disitamab vedotin in biomarker-non-enriched upper tract urothelial carcinoma

**DOI:** 10.3389/fonc.2026.1857919

**Published:** 2026-09-03

**Authors:** Guohui Cheng, Quan Zhang, Rundong Li, Zhongze Zhou, Xiangbin Kong, Zhilong Dong

**Affiliations:** 1 Department of Urology, The Second Hospital and Clinical Medical School, Lanzhou University, Lanzhou, Gansu, China; 2 Institute of Urology, Gansu Province Clinical Research Center for Urinary System Diseases, The Second Hospital of Lanzhou University, Lanzhou, Gansu, China

**Keywords:** biomarker-non-enriched, case report, disitamab vedotin, glandular differentiation, toripalimab, upper tract urothelial carcinoma

## Abstract

Upper tract urothelial carcinoma (UTUC) is uncommon and aggressive, and evidence for perioperative antibody-drug conjugate or immunotherapy remains limited. We report a 59-year-old man with biomarker-non-enriched high-risk ureteral UTUC treated with four cycles of toripalimab plus disitamab vedotin before planned surgery. Magnetic resonance urography, urine cytology, and ureteroscopic biopsy supported high-grade UTUC with glandular differentiation; CDX-2, villin, and β-catenin positivity prompted exclusion of a gastrointestinal primary. The biopsy showed HER2 0, TROP2 0, and low PD-L1 expression. Restaging MRI and endoscopy demonstrated reduced visible lesion extent; axial dimensions decreased from 3.68 × 2.69 cm to 2.14 × 1.55 cm. No formal RECIST 1.1 category was assigned because imaging was not standardized. The patient then underwent planned laparoscopic radical nephroureterectomy with bladder cuff excision. Final pathology showed poorly differentiated ureteral urothelial carcinoma with marked histological heterogeneity, staged ypT2Nx. Recovery was uneventful. At the latest follow-up on May 28, 2026, the patient remained well, and surveillance cystoscopy showed no evident intravesical abnormality. This case highlights the limited representativeness of pretreatment biopsy in UTUC. The observed lesion reduction is hypothesis-generating and should not be interpreted as evidence of clinical efficacy. Standardized response assessment and longer follow-up are required.

## 1 Introduction

Upper tract urothelial carcinoma (UTUC) is an uncommon but aggressive malignancy, accounting for 5–10% of all urothelial carcinomas, and radical nephroureterectomy with bladder cuff excision remains the standard treatment for high-risk non-metastatic disease (1, 2). Although perioperative platinum-based therapy is increasingly used in selected patients, evidence for perioperative immunotherapy- or antibody-drug conjugate (ADC)-based strategies in UTUC remains limited (2). In that setting, disitamab vedotin (DV), alone or in combination with toripalimab, has shown activity beyond biomarker-enriched populations, including human epidermal growth factor receptor 2 (HER2)-unselected, HER2-negative/HER2-low, and unselected urothelial carcinoma settings (3–5). In parallel, histological subtypes or divergent differentiation in UTUC are associated with more aggressive clinicopathological features and poorer outcomes (6), whereas preoperative biopsy may undergrade, understage, or incompletely reflect the overall tumor phenotype (7). Against this background, we report a clinically localized and surgically resectable high-risk ureteral UTUC with a diagnostically challenging immunophenotype requiring exclusion of a gastrointestinal primary. Despite a biomarker-non-enriched profile in the pretreatment biopsy, characterized by concurrent HER2 0, trophoblast cell-surface antigen 2 (TROP2) 0, and low programmed death-ligand 1 (PD-L1) expression, a reduction in the visible lesion extent was observed after four cycles of toripalimab plus DV before the patient underwent the planned radical nephroureterectomy.

## 2 Case description

### 2.1 Patient information

In November 2025, a 59-year-old man presented with 5 days of painless gross hematuria. He had no significant past medical history, prior urologic intervention, regular medication use, known drug allergies, relevant family history, or identifiable psychosocial or genetic factors related to the present disease. No relevant diagnostic or therapeutic intervention had been performed before the current presentation. Preoperative evaluation suggested a clinically localized high-risk left ureteral tumor without definite nodal or distant metastasis.

### 2.2 Diagnostic assessment

**Figure 1:**
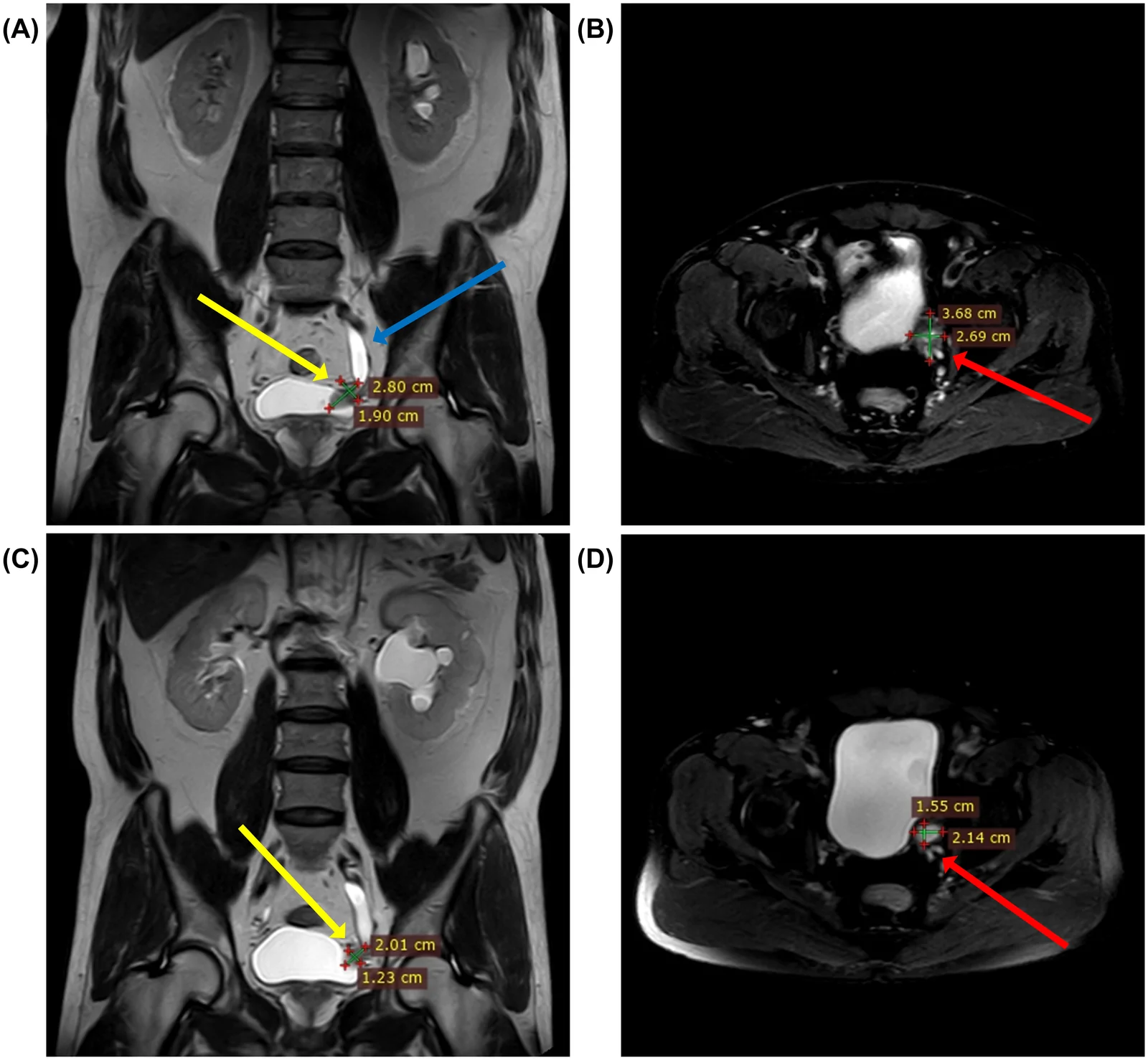
Magnetic resonance imaging before and after systemic therapy. **(A)** Pretreatment coronal image showing the distal left ureteral lesion measuring 2.80 × 1.90 cm. **(B)** Pretreatment axial image showing lesion dimensions of 3.68 × 2.69 cm. **(C)** Post-treatment coronal image showing reduction to 2.01 × 1.23 cm. **(D)** Post-treatment axial image showing reduction to 2.14 × 1.55 cm. Measurements were obtained retrospectively on comparable imaging planes; no formal RECIST 1.1 response category was assigned.

**Figure 2:**
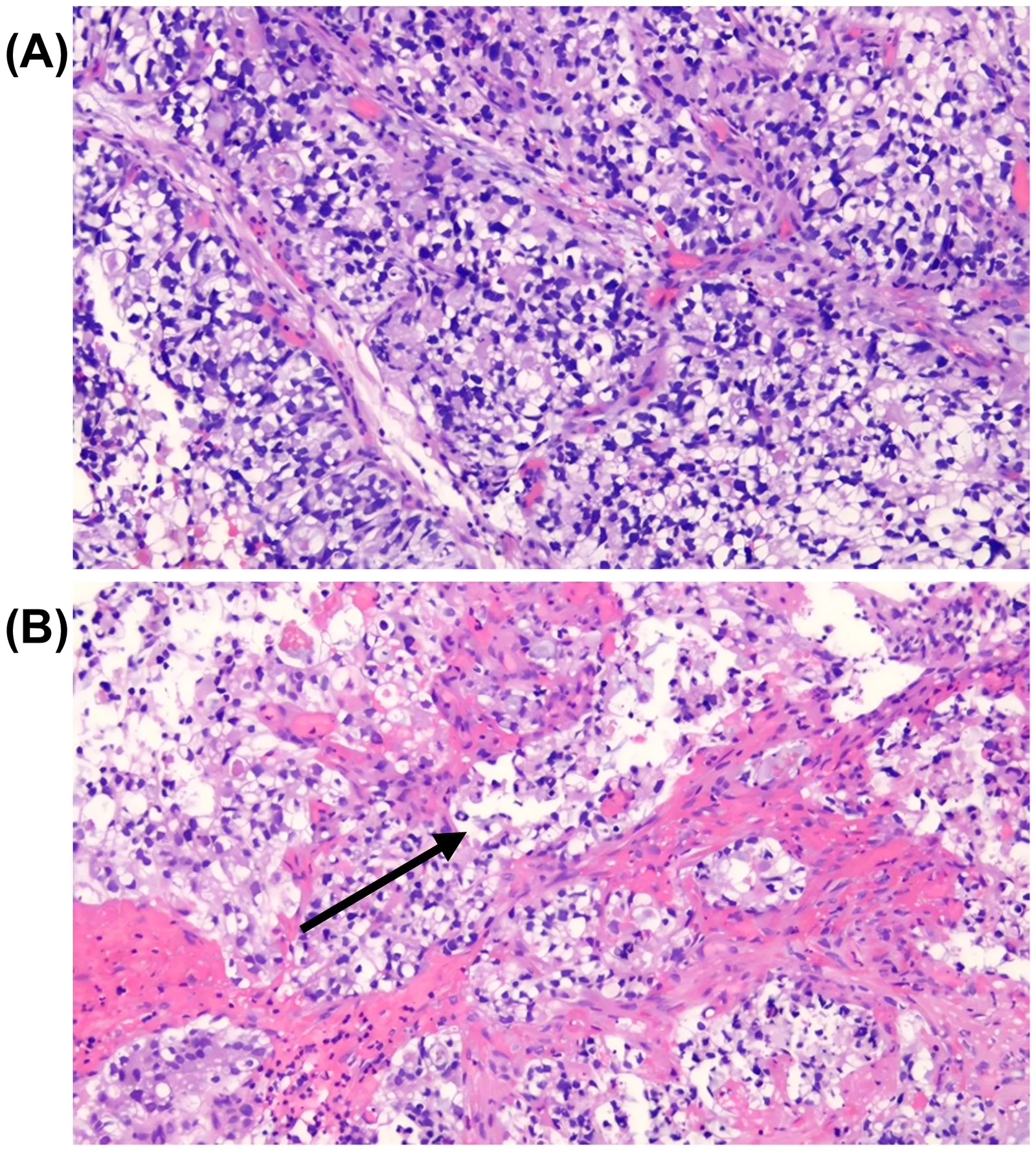
Pretreatment ureteroscopic biopsy pathology. **(A, B)** Hematoxylin-eosin staining showed a high-grade malignant epithelial neoplasm with infiltrative growth and glandular/adenocarcinoma-like morphological features, raising the differential diagnosis of urothelial carcinoma with glandular differentiation versus a gastrointestinal primary.

Magnetic resonance urography showed a long-segment lesion in the mid-to-distal left ureter, with luminal narrowing, ureteral wall thickening, and upstream dilatation of the left ureter and collecting system (**Figures 1A, B**). Urine cytology revealed atypical urothelial cells. Cystoscopy and ureteroscopy showed no obvious intravesical mass but identified a bulging lesion at the left ureteral orifice and an approximately 10-cm intraluminal lesion in the left ureter. As complete endoscopic removal was not feasible, partial endoscopic resection was performed for histopathological evaluation. The initial pathological diagnosis favored high-grade urothelial carcinoma with glandular differentiation. Hematoxylin-eosin staining showed a high-grade malignant epithelial neoplasm with infiltrative growth and glandular/adenocarcinoma-like morphology (**Figures 2A, B**). The pretreatment specimen showed null-pattern p53 expression, reported as consistent with a TP53 nonsense mutation, and a hotspot Ki-67 labeling index of approximately 50%. Positivity for CDX-2, villin, and β-catenin raised the differential diagnosis of a gastrointestinal primary. The pretreatment biopsy showed a biomarker-non-enriched profile, with HER2 0, TROP2 0, and low PD-L1 expression by the Ventana SP263 assay (<25% of tumor cells and <1% of immune cells).

### 2.3 Therapeutic intervention

Radical nephroureterectomy remained the planned definitive treatment throughout the clinical course. Before systemic treatment, the patient had an Eastern Cooperative Oncology Group performance status of 1 and a serum creatinine level of 84.3 μmol/L. Although the available clinical findings did not establish formal consensus-defined cisplatin ineligibility, the treating team considered that the obstructive upper urinary tract changes and concurrent urinary inflammatory findings raised concerns about the risk–benefit balance of cisplatin-based treatment. This assessment took into account the long-segment obstructive ureteral lesion with upstream ureteral and collecting-system dilatation, marked pyuria and leukocyte esterase positivity suggestive of concurrent urinary tract inflammation or infection, and concern regarding cisplatin-associated nephrotoxicity and the requirement for intensive hydration.

The treatment decision was made by the treating urology team rather than through a formal multidisciplinary conference, after review of the imaging and pathological findings and discussion of the proposed strategy with the patient. The approximately 10-cm high-grade ureteral lesion, glandular differentiation, limited pretreatment biopsy tissue, and concern regarding occult micrometastatic disease supported consideration of systemic treatment before surgery. The objective was to obtain preoperative disease control and address potential micrometastatic disease before the planned radical nephroureterectomy, rather than to convert the tumor to resectability or reduce the planned surgical extent.

Given the emerging clinical activity of disitamab vedotin plus toripalimab in HER2-unselected advanced urothelial carcinoma (3), together with the recognized limitations of biomarker assessment on a small ureteroscopic biopsy, the combination was selected as an individualized, non-standard preoperative regimen. The biomarker-non-enriched profile was not considered to provide a validated basis for treatment selection. The patient was informed that the regimen was not an established perioperative standard for localized UTUC and that the supporting evidence was primarily derived from advanced urothelial carcinoma. After providing informed consent, he received four cycles of toripalimab (240 mg) plus disitamab vedotin (2.0 mg/kg; 120 mg for a body weight of 60 kg) without obvious treatment-related adverse events. In March 2026, he underwent the planned laparoscopic left radical nephroureterectomy with bladder cuff excision.

### 2.4 Follow-up and outcomes

**Figure 3:**
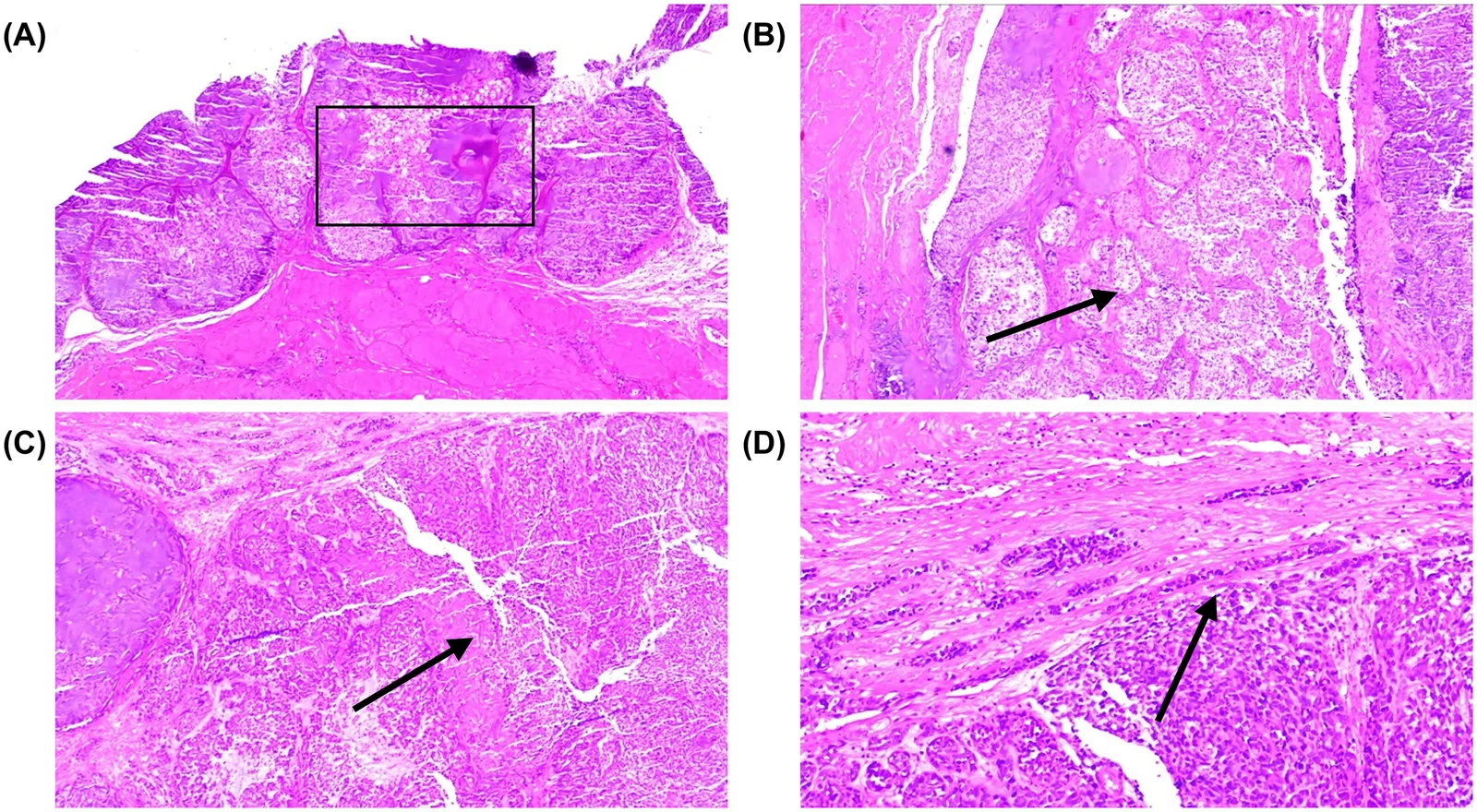
Postoperative histopathology of the resection specimen. **(A)** Low-power view showing invasive ureteral urothelial carcinoma with complex architectural heterogeneity; the boxed area highlights a representative tumor region. **(B)** Tumor areas with myxoid- and chordoid-like morphology. **(C)** Additional heterogeneous component showing solid/high-grade urothelial carcinoma architecture within fibrotic stroma. **(D)** Tumor infiltration in association with stromal reaction and wall involvement. (Hematoxylin-eosin staining).

**Figure 4:**
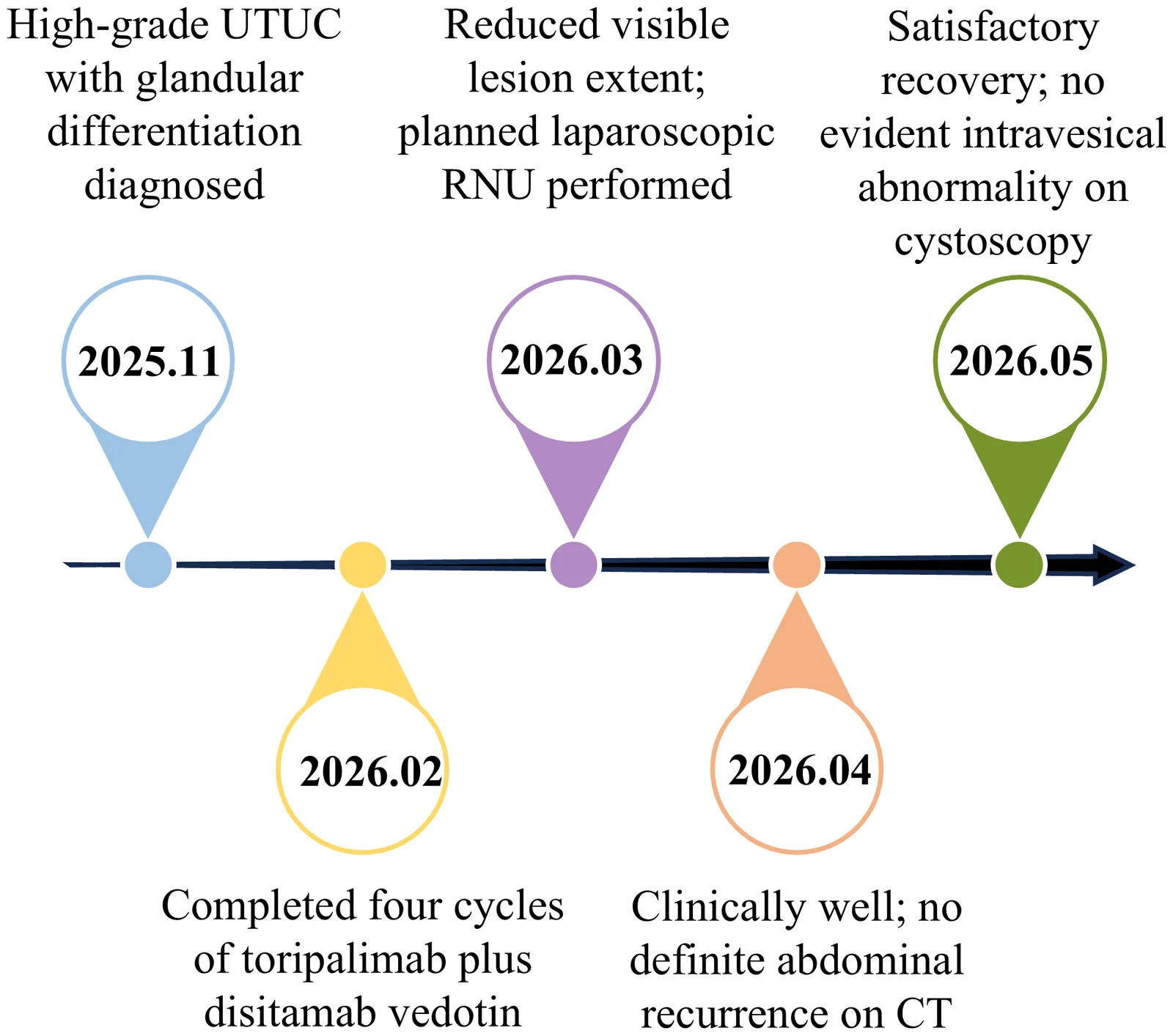
Clinical timeline of diagnosis, preoperative systemic treatment, planned radical nephroureterectomy, and postoperative follow-up. UTUC, upper tract urothelial carcinoma; RNU, radical nephroureterectomy; CT, computed tomography.

Retrospective comparison of the pretreatment and post-treatment magnetic resonance images showed that the maximal measurable dimensions of the distal ureteral lesion decreased from 3.68 × 2.69 cm to 2.14 × 1.55 cm on axial images and from 2.80 × 1.90 cm to 2.01 × 1.23 cm on coronal images (**Figures 1A–D**). The residual visible abnormality was predominantly confined to the distal ureter. Because the lesion showed irregular ureteral-wall involvement and imaging was not standardized for response assessment, no formal RECIST 1.1 response category was assigned. Follow-up endoscopy showed reduced exophytic tumor burden with residual irregular change at the lesion site, and repeat urine cytology showed no evidence of high-grade urothelial carcinoma or non-urothelial malignancy. Final pathology demonstrated poorly differentiated ureteral urothelial carcinoma with marked histological heterogeneity, predominantly showing myxoid- and chordoid-like features, together with minor sarcomatoid and high-grade conventional urothelial components, focal glandular differentiation, and focal neuroendocrine differentiation (**Figures 3A–D**). The resection specimen again showed null-pattern p53 expression, and the Ki-67 labeling index was approximately 80%. The post-treatment pathological stage was ypT2Nx. The renal parenchyma, renal capsule, renal pelvis, and vas deferens were free of tumor invasion, whereas urothelial carcinoma *in situ* was present in the adjacent urothelium and closely approached the bladder cuff margin. The postoperative course was uneventful, and the patient was discharged 7 days after surgery. At the initial postoperative follow-up in April 2026, the patient remained in good general condition without new symptoms or complaints. Non-contrast abdominal CT demonstrated postoperative changes at the left nephroureterectomy bed, with mild local exudative/partially encapsulated change and slight adjacent psoas swelling, without definite enlarged retroperitoneal lymph nodes. Chest radiography revealed no significant abnormality. At the latest follow-up on May 28, 2026, the patient reported satisfactory recovery without obvious discomfort, and surveillance cystoscopy showed no evident intravesical abnormality. The available follow-up assessments showed no definite evidence of intravesical or abdominal recurrence. However, the overall observation period remained short, and continued cystoscopic and systemic surveillance was planned. The overall clinical course is summarized in **Figure 4**.

## 3 Discussion

This case highlights three clinically relevant issues in UTUC: the limited representativeness of ureteroscopic biopsy, the cautious interpretation of an observed reduction in lesion extent after combined ADC-immunotherapy in a biomarker-non-enriched setting, and the clinical significance of marked histological heterogeneity in the resection specimen.

First, the discrepancy between the initial biopsy and the final nephroureterectomy specimen should be interpreted in light of the recognized limitations of preoperative sampling in UTUC. For surgically eligible high-risk disease, radical nephroureterectomy with bladder cuff excision remains the standard treatment, yet pretreatment decision-making still relies heavily on imaging, ureteroscopic findings, and limited biopsy material (2). A systematic review and meta-analysis showed that ureteroscopic biopsy undergraded and understaged UTUC in 32% and 46% of cases, respectively, with a negative predictive value of only 60% for apparently non-muscle-invasive disease (7). Therefore, a small biopsy capturing only a glandular-appearing or biomarker-low component cannot be assumed to represent the whole tumor. This is particularly relevant in the present case, in which the nephroureterectomy specimen demonstrated marked histological heterogeneity. Histological subtypes or divergent differentiation in UTUC are consistently associated with more aggressive clinicopathological features and worse outcomes (6). A recent meta-analysis of 14,407 patients treated with radical nephroureterectomy likewise showed higher rates of adverse pathological features and significantly poorer cancer-specific, overall, and recurrence-free survival in tumors with histological subtypes/divergent differentiation than in pure urothelial carcinoma (6).

Second, the initial diagnostic difficulty in this patient was pathologically plausible rather than exceptional. Urothelial carcinoma with glandular differentiation may overlap morphologically and immunophenotypically with enteric-type adenocarcinoma, and no small immunohistochemical panel is fully definitive in isolation (8). In a comparative immunohistochemical study that included urothelial carcinoma with glandular differentiation, Rao et al. found that nuclear GATA3 staining was present in only a minority of such cases, whereas p63 labeled the urothelial but not the glandular component, and β-catenin staining patterns were helpful in distinguishing these lesions from colorectal adenocarcinoma (8). Accordingly, positivity for enteric-associated markers such as CDX-2, villin, and β-catenin should prompt careful exclusion of a gastrointestinal primary, but should not automatically exclude urothelial carcinoma with glandular differentiation. In the present case, the resection specimen clarified the diagnosis as a highly heterogeneous urothelial carcinoma rather than a purely enteric-type malignancy, illustrating how sampling bias can affect both morphological interpretation and biomarker readout in UTUC.

Third, the biomarker findings should be interpreted as a non-enriched profile in the sampled pretreatment biopsy rather than as definitive evidence of uniformly absent or low expression throughout the tumor. Ureteroscopic biopsy samples only a small fraction of a long-segment lesion, whereas the nephroureterectomy specimen in this case demonstrated multiple divergent histological components, providing direct evidence of histological intratumoral heterogeneity. Spatial analyses of muscle-invasive bladder cancer have shown regional variation in HER2 and TROP2 expression (9), while PD-L1 results may differ between limited samples and larger resection specimens because of heterogeneous staining patterns (10). Therefore, HER2 0, TROP2 0, and low PD-L1 expression in a single biopsy may reflect sampling of a low-expression region rather than the biomarker status of the entire tumor. This possibility remains unproven in the present case because multiregional biomarker testing of the nephroureterectomy specimen was not performed. Disitamab vedotin comprises a HER2-directed antibody conjugated to MMAE through a cleavable linker; preclinical findings suggest that this structure may permit bystander killing of adjacent tumor cells with low or heterogeneous HER2 expression (11). Clinical activity of disitamab vedotin-based therapy has also been reported in HER2-unselected and HER2-negative/HER2-low urothelial carcinoma populations (3, 4, 12). Low PD-L1 expression is likewise a relative rather than absolute negative predictor of response to PD-1 blockade (5). TROP2 is not the direct target of either disitamab vedotin or toripalimab and was included only as a descriptive component of the biomarker profile. Thus, the observed reduction in lesion extent may reflect sampling-related biomarker misclassification, spatially heterogeneous target expression, ADC-mediated bystander killing, and/or the contribution of PD-1 blockade. However, none of these mechanisms was directly tested in this case, and the observation does not establish biomarker-independent efficacy.

Finally, the postoperative pathology further supports the aggressive and heterogeneous biology of this tumor. The coexistence of multiple variant histologic components, including sarcomatoid and glandular differentiation, is concordant with previous reports linking variant histology in UTUC to advanced stage and worse prognosis (6, 13, 14). The minor sarcomatoid component is also notable, because sarcomatoid urothelial carcinoma of the upper tract is rare, usually presents at an advanced stage, and has historically been associated with poor outcomes (15). Emerging retrospective evidence has suggested a potential role for definitive primary-tumor treatment as part of multimodal management in selected patients with advanced UTUC. In a National Cancer Database analysis of stage IV UTUC, high-intensity local treatment of the primary tumor was associated with longer overall survival (16). A subsequent systematic review and meta-analysis of metastatic UTUC similarly found that systemic therapy followed by consolidative surgery, as well as cytoreductive surgery combined with systemic therapy, was associated with improved overall survival compared with non-surgical management (17). However, these findings were derived predominantly from retrospective, highly selected cohorts and remain susceptible to selection bias. They support further investigation of integrating systemic and definitive local treatments but cannot be directly extrapolated to the present patient, whose disease was localized and surgically resectable at baseline. Nevertheless, this case should not be overinterpreted. Radical nephroureterectomy remained the planned definitive treatment, and the preoperative regimen should not be interpreted as having converted an unresectable tumor to resectability or as having enabled surgery. The non-platinum regimen was selected on an individualized risk–benefit basis because the treating team considered cisplatin-associated nephrotoxicity and intensive hydration unfavorable in the context of obstructive upper urinary tract disease and concurrent urinary inflammation, rather than because the patient met formal criteria for cisplatin ineligibility. Instead, the present case documents an observed association between preoperative toripalimab plus disitamab vedotin and a reduction in lesion extent before planned surgery. A major strength of this report is the integrated clinicopathological correlation across pretreatment biopsy, restaging imaging, endoscopic findings, and final surgical pathology. However, its limitations include the single-case design, absence of multiregional molecular testing, inability to distinguish the individual contributions of DV and toripalimab, retrospective imaging measurements without formal RECIST 1.1 assessment, lack of standardized pathological response assessment, and absence of formal pathological nodal evaluation. The postoperative observation period extended only from radical nephroureterectomy in March 2026 to the latest assessment on May 28, 2026. Although surveillance cystoscopy at the latest follow-up showed no evident intravesical abnormality and the April 2026 abdominal CT showed no definite evidence of local recurrence or abdominal metastatic disease, this limited interval is insufficient to assess durable disease control or exclude subsequent bladder recurrence, local relapse, distant metastasis, or delayed treatment-related toxicity. Continued long-term cystoscopic and systemic surveillance is therefore required.

## 4 Patient perspective

Before the diagnosis and treatment were completed, I felt worried about my condition and the uncertainty surrounding the disease. After systemic treatment and surgery, I recovered well without obvious discomfort, and I was satisfied with the overall treatment process and the current outcome. At follow-up, I remained in good condition. I hope that sharing my experience may help improve the understanding and management of similar patients.

## 5 Conclusion

This case illustrates that limited ureteroscopic biopsy may not fully capture the pathological and biomarker heterogeneity of UTUC. In this patient with clinically localized, resectable disease and HER2 0, TROP2 0, and low PD-L1 expression, visible lesion extent decreased after four cycles of toripalimab plus disitamab vedotin before planned radical nephroureterectomy. This treatment should not be interpreted as enabling surgery. Given the single-case design, short follow-up, inability to separate the effects of the two agents, and non-standardized response assessment, this observation is hypothesis-generating and does not establish clinical efficacy. Further studies with standardized assessment and longer follow-up are required.

## Data Availability

The original contributions presented in the study are included in the article/supplementary material. Further inquiries can be directed to the corresponding author.
